# A retrospective analysis from NHANES 2003–2018 on the associations between inflammatory markers and coronary artery disease, all-cause mortality and cardiovascular mortality

**DOI:** 10.1371/journal.pone.0326953

**Published:** 2025-07-09

**Authors:** Tong Sun, Penglei Chen, Xuwei Zheng

**Affiliations:** 1 Department of Cardiology, Suzhou Hospital of Anhui Medical University, Suzhou, Anhui, China; 2 Department of cardiology, the First Affiliated Hospital of Zhengzhou University, Zhengzhou, Henan, China; Iuliu Hațieganu University of Medicine and Pharmacy: Universitatea de Medicina si Farmacie Iuliu Hatieganu, ROMANIA

## Abstract

**Background:**

The objective of this research was to investigate the associations between inflammation markers and coronary artery disease (CAD), along with all-cause mortality and cardiovascular mortality.

**Methods:**

This study utilized data from the National Health and Nutrition Examination Survey (NHANES) collected between 2003 and 2018. The platelet-to-lymphocyte ratio (PLR), neutrophil-to-lymphocyte ratio (NLR), monocyte-to-lymphocyte ratio (MLR), and systemic immune inflammation index (SII) were calculated based on blood test results. The diagnosis of CAD was obtained from self-reported cardiovascular health questionnaires. Participants’ survival status was sourced from the National Death Index (NDI) of the National Center for Health Statistics (NCHS). Logistic and Cox regression models were employed to investigate the associations between PLR, NLR, MLR, and SII with CAD, all-cause mortality, and cardiovascular mortality.

**Results:**

A total of 32,683 individuals from the 2003–2018 NHANES were involved. After adjusting for potential confounders, each 1-unit increase in log (NLR) and log (MLR) was associated with a 29% (95% CI: 1.15–1.46, *P* < 0.001) and 67% (95% CI: 1.40–1.99, *P* < 0.001) increase in the risk of CAD, respectively. Notably, when log (PLR) exceeded 4.93(PLR = 138.38) and log (SII) surpassed 6.11(SII = 450.34), the risk of CAD increased sharply (*P* < 0.001). Furthermore, individuals in the highest quartiles (Q4) of PLR, NLR, MLR, and SII had significantly higher risks of all-cause mortality (13%, 88%, 91%, and 42%, respectively) and cardiovascular mortality (48%, 194%, 139%, and 90%, respectively) compared to those in the lowest quartile (Q1), with all *P*-values <0.001. Moreover, MLR had the highest the area under the curve (AUC) value (AUC:0.642, 95% CI: 0.629–0.654), followed by NLR (AUC:0.600, 95% CI: 0.587–0.612) for distinguishing CAD.

**Conclusion:**

In this study, we found that PLR, NLR, MLR, and SII were associated with increased prevalence of CAD, as well as increased risks of all-cause and cardiovascular mortality. These inflammatory markers may serve as valuable clinical indicators for CAD, all-cause and cardiovascular mortality in the general population.

## Introduction

Coronary artery disease (CAD) continues to be a significant contributor to global mortality and disability, presenting a substantial health challenge on a worldwide scale. From 1990 to 2019, the number of deaths due to CAD in the world has consistently risen from 12.1 million to 18.6 million [1,2]. This increasing trend underscores the critical importance of implementing efficient prevention, early detection, and management measures to combat the escalating impact of CAD on public health.

Although numerous risk factors for CAD are well-established, further research is needed to better understand their relationship with inflammation. Evidence suggests that the immune-inflammatory response plays a key role in the onset and progression of CAD. It is crucial to emphasize the clinical significance of inflammation biomarkers in the early diagnosis, monitoring of the disease, evaluation of treatment efficacy, and prognosis assessment of CAD [3], which is considered as a chronic inflammation-related disease characterized by a prolonged non-infectious inflammatory state that disrupts the body’s equilibrium and is associated with various medical conditions like diabetes mellitus, cancer, and metabolic syndrome [4]. Originally serving as a protective mechanism against infections and aiding in tissue repair after injury, chronic inflammation has been shown to have detrimental effects, particularly in disease states [5–7]. Inflammation is a dynamic response triggered by immune and non-immune cells in response to external factors and diseases. Macrophages and T lymphocytes contribute to adverse cardiovascular outcomes by promoting chronic inflammation and vascular remodeling through the release of pro-inflammatory cytokines, such as interleukin-6 (IL-6), and the activation of systemic inflammatory markers like C-reactive protein (CRP), which are associated with a higher risk of atherosclerotic plaque rupture and cardiovascular events [8,9]. Given the intricate nature of this response, composite biomarkers like the Platelet to Lymphocyte Ratio (PLR), Neutrophil to Lymphocyte Ratio (NLR), Monocyte to Lymphocyte Ratio (MLR), and the Systemic Immune Inflammation Index (SII) are useful for more comprehensive and effective prediction. The SII, a recently discovered marker for inflammation linked to poor outcomes in different types of cancers [10,11], is calculated using platelet, neutrophil, and lymphocyte counts to assess a patient’s inflammatory and immune status. As such, the SII may serve as a valuable prognostic tool for various malignant diseases.

Prior studies have extensively examined the link between inflammatory hematological markers and CAD, all-cause mortality, as well as cardiovascular mortality in patients suffering from a chronic inflammation-related disease [6,7]. Nevertheless, data is lacking regarding the importance of these inflammatory markers within the general population. The relationships among PLR, NLR, MLR, and SII with CAD, all-cause mortality, and cardiovascular mortality in the general populace remain unclear. Chronic inflammation is commonly seen in individuals due to factors including significant life stress, poor lifestyle choices, late night activities, substandard living conditions, and the aging process [12]. Although chronic inflammation might not cause chronic diseases, individuals experiencing chronic inflammation are at an increased risk of developing chronic diseases such as CAD. Thus, this study set out to assess the predictive value of PLR, NLR, MLR, and SII with regard to CAD, all-cause mortality, and cardiovascular mortality in the general population by utilizing data from a large-scale public database.

## Methods

### Study participant

The National Health and Nutrition Examination Survey (NHANES) was a comprehensive nationwide survey overseen by the Centers for Disease Control and Prevention (CDC) [13]. Conducted with approval from the Research Ethics Review Board of the National Center for Health Statistics (NCHS), the survey aimed to gather a wide range of information on demographic, socioeconomic, and health-related topics through interviews, physical examinations, and laboratory tests on biological samples. This detailed survey employs sophisticated sampling techniques to select participants, and oversampling specific populations to ensure a representative sample. Upon obtaining informed consent, demographic and questionnaire data were collected during household interviews conducted by trained technicians. Subsequent health screenings and biospecimen collection were conducted at mobile examination centers (MEC) following the household interviews. The meticulous data collection methods and procedures of the survey were elaborately outlined on the official website. Validation of all NHANES data was done by the NCHS, with updates being provided biennially on the website (https://wwwn.cdc.gov/nchs/nhanes/default.aspx.).

To clarify, our study utilized data from NHANES participants from 2003 to 2018 (n = 80,312), and this was secondary use of data. Exclusion criteria were applied to individuals under the age of 20(n = 35,522), those with missing PLR, NLR, MLR, and SII data(n = 8600), as well as those lacking mortality and CAD information(n = 3507). Our study’s data can be accessed in the supplementary materials.

### Data collection and definitions

Interviews conducted at households aimed to collect information on participants’ demographic characteristics, and habits related to smoking and alcohol consumption, as well as their medical history, which included the use of prescribed medications. Physical measurements such as height, weight, waist circumference, and blood pressure were performed at the Mobile Examination Center (MEC). Before giving blood samples for analysis, participants were instructed to fast for 8 hours; these samples were subsequently sent to a partner laboratory for tests including liver and kidney function, complete blood count, blood glucose, and lipid profiles. As a result, we were able to obtain laboratory data for participants, including glycosylated hemoglobin type A1C(HbA1c), fasting blood glucose (FBG), total cholesterol (TC), triglycerides (TG), high-density lipoprotein cholesterol (HDL-C), and low-density lipoprotein cholesterol (LDL-C).

Specific mathematical formulas were used to carry out the calculations for PLR, NLR, MLR, and SII [14].


PLR= Platelet count/ Lymphocyte count\]

NLR= Neutrophil count/ Lymphocyte count\]

MLR= Monocyte count/ Lymphocyte count\]

SII= Platelet count × Neutrophil count/ Lymphocyte count\]

Smoking behavior was classified as non-smokers (fewer than 100 cigarettes smoked in the lifetime), former smokers (at least 100 cigarettes smoked in the lifetime and quit for more than one year), and current smokers (at least 100 cigarettes smoked in the lifetime and still smoking) [15]. Correspondingly, alcohol consumption was categorized into non-drinkers, those who consumed alcohol 1–5 times per month, 5–10 times per month, or more than 10 times per month [16].

### Outcome assessment

The study’s primary outcome was the prevalence of CAD, with the secondary outcome for all-cause mortality and cardiovascular mortality. In this research, the determination of CAD diagnosis relied on the self-reported medical histories of the participants. Participants were inquired about whether they had ever been diagnosed with coronary heart disease, angina, or a heart attack by a professional physician. Those who answered positively to any of these conditions were classified as having CAD. The NCHS matched the survival status data of NHANES participants in the National Death Index (NDI) using Social Security numbers and birth dates. After processing by trained personnel, the survival data of NHANES participants were published on the official website, including survival status, follow-up time, cause of death, and other details. NHANES participants were assigned a unique identifier upon entering the study, which allowed the integration of mortality data with baseline examination data using this unique code. The follow-up period for participants begins from the date of participation and ends on December 31, 2019 (the most recent date when the NCHS published mortality data). If a participant dies during this period, the follow-up time will be calculated from the date of participation to the date of death. If a participant is still alive at the end of the follow-up period, the follow-up time will be calculated from the date of participation to the end of the follow-up period (December 31, 2019) [13].

### Statistical analysis

Statistical analyses were conducted using R software version 4.3.0. Participants with a small amount of missing data (missing rate <10%) on poverty income ratio (PIR), body mass index (BMI),  TG, FBG, HbA1C, HDL-C, LDL-C, smoking status, and alcohol consumption were imputed using the random forest method in the mice package in R.4.3.0. Sample weights were applied to reduce sampling bias, and following the NHANES analytical guidelines, new weights were recalculated by dividing the original 2-year cycle weights (WTMEC2YR) by 8 (the number of cycles). As PLR, NLR, MLR, and SII exhibited skewed distributions, log transformation was applied to normalize these variables for subsequent analyses. Notably, an increase of one unit in the log-transformed independent variable corresponded to a 2.72-fold increase in the original value. Additionally, these markers were categorized into quartiles to further assess their association with outcomes. Continuous variables following a normal distribution were presented as mean ± standard deviation (SD), categorical variables were summarized as percentages. For group comparisons, T-tests were employed for continuous variables, and chi-square tests were used for categorical variables. Before conducting the logistic regression analysis, we assessed multicollinearity among independent variables using the variance inflation factor (VIF). We calculated the VIF for all covariates and iteratively removed variables with VIF ≥ 5 to ensure model stability and interpretability. Ultimately, all covariates included in the final model had VIF < 5, thereby reducing the impact of multicollinearity on regression estimates [17]. The proportional hazards (PH) assumption was tested for all covariates to confirm the suitability of the Cox regression model.

Multifactorial logistic regression models were employed to evaluate the associations of PLR, NLR, MLR, and SII with CAD, presenting odds ratios (ORs) with corresponding 95% confidence intervals (CIs). Kaplan-Meier analysis was used to assess the cumulative incidence of all-cause death and cardiovascular death. To further investigate the association between PLR, NLR, MLR, SII, and all-cause along with cardiovascular mortality, multifactorial Cox regression models were applied, reporting hazard ratios (HRs) and associated 95% CIs. Model 1 was unadjusted, while Model 2 was adjusted for demographic and socioeconomic factors (gender, age, race, education, PIR, and BMI). Model 3 further incorporated clinical laboratory markers (HbA1c, TG, HDL-C, LDL-C, FBG) and lifestyle factors (smoking and alcohol consumption)), alongside the variables in Model 2. Restricted cubic spline (RCS) regression analysis was employed to examine the nonlinearity and dose-response relationship between PLR, NLR, MLR, SII, and CAD, all-cause mortality, cardiovascular mortality. Nonlinearity was evaluated through a likelihood ratio test, and when a significant nonlinear relationship was observed, a two-stage segmented regression analysis was applied to explore the predictor’s threshold effect on the outcome by identifying the inflection point. The discriminative ability for CAD of PLR, NLR, MLR, or SII were assessed using receiver operating characteristic (ROC) curves, with the area under the curve (AUC) and corresponding 95% CIs. To confirm the robustness of the results, sensitivity analyses were performed. Initially, the associations between PLR, NLR, MLR, SII, and CAD were reanalyzed after excluding participants with missing data. Subsequently, multiple imputations were conducted, generating 10 iterations to produce 10 complete datasets, in which the relationships between PLR, NLR, MLR, SII, and CAD were systematically reassessed. The 95% CIs for ORs, HRs, and AUC were inclusive of both endpoints within parentheses. In this study, A two-sided *P*-value < 0.05 was considered statistically significant.

## Results

### Clinical characteristics of the study population

**Fig 1:**
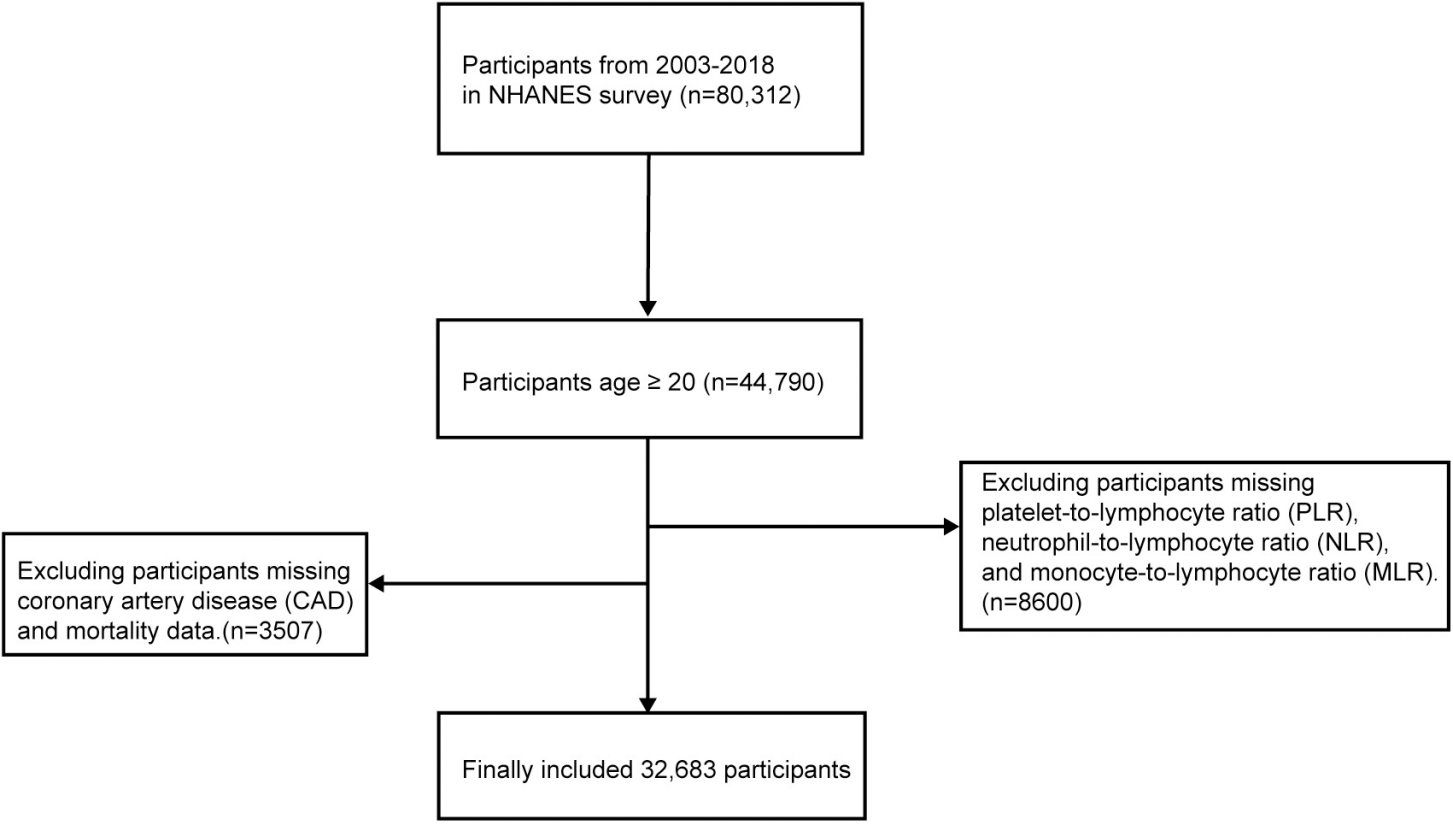
Flow chart for the enrollment of study population.

**Fig 2:**
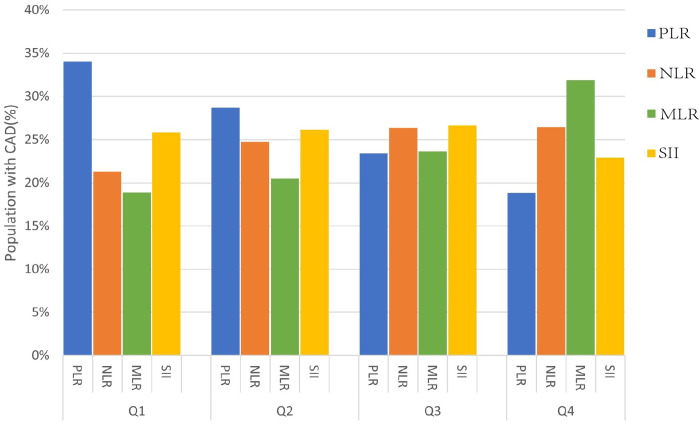
The proportion of patients with CAD sorted by quartiles of PLR, NLR, MLR, SII. PLR, platelet-to-lymphocyte ratio; NLR, neutrophil-to-lymphocyte ratio; MLR, monocyte-to-lymphocyte ratio; SII, the systemic immune inflammation index; CAD, coronary artery diseases; Q, quartiles.

In this investigation, a total of 32,683 individuals from the 2003–2018 NHANES were involved (Fig 1). The variances in clinical characteristics between the CAD and non-CAD groups were summarized in Table 1, indicated that those in the CAD cohort were more likely to be of non-Hispanic white descent, male, older, with lower educational attainment, smokers(*P* < 0.001). During median follow-up of 97 months, subjects diagnosed with CAD exhibited a higher rate of all-cause mortality (36.5% vs. 9.7%) and cardiovascular mortality (12.8% vs. 2.1%), along with high levels of FBG, TG, and HbA1c(*P* < 0.001). Moreover, participants with CAD demonstrated higher values of NLR, MLR and SII compared to participants without CAD(*P* < 0.001). The distribution of participants with CAD based on PLR, NLR, MLR, and SII quartiles was depicted in Fig 2. High quartiles of NLR and MLR were linked to a higher prevalence of CAD, whereas increased quartiles of PLR were associated with lower CAD prevalence. Conversely, SII quartiles showed similar proportions of CAD cases.

**Table 1:** The characteristics of participants according to CAD.

Variable	Overall	Non-CAD	CAD	*P value*
(n = 32683)	(n = 30299)	(n = 2384)	
Female (%)	16876(51.6)	15969(52.7)	907(38.0)	<0.001
Age (years)				<0.001
20-40	11096(34.0)	11009(36.3)	87(3.6)	
40-60	10751(32.9)	10261(33.9)	490(20.6)	
>60	10836(33.2)	9029(29.8)	1807(75.8)	
Education (%)				<0.001
Less than high school	8360(25.6)	7547(24.9)	813(34.1)	
High school graduate	7462(22.8)	6883(22.7)	579(24.3)	
Some college or above	16861(51.6)	15869(52.4)	992(41.6)	
Race (%)				<0.001
Mexican American	5241(16.0)	4998(16.5)	243(10.2)	
Non-Hispanic White	14112(43.2)	12741(42.1)	1371(57.5)	
Non-Hispanic Black	6771(20.7)	6349(21.0)	422(17.7)	
Other/multiracial	6559(20.1)	6211(20.5)	348(14.6)	
PIR	2.50(1.62)	2.52(1.63)	2.18(1.49)	<0.001
BMI (kg/m2)	29.14(6.93)	29.06(6.92)	30.20(6.92)	<0.001
TC (mg/dl)	193.94(42.13)	195.26(41.59)	177.17(45.29)	<0.001
TG (mg/dl)	154.79(124.33)	153.75(124.94)	168.00(115.48)	<0.001
HDL-C (mg/dl)	53.14(16.30)	53.44(16.33)	49.33(15.46)	<0.001
LDL-C (mg/dl)	113.33(35.68)	114.39(35.37)	99.86(36.90)	<0.001
HbA1c	5.75(1.09)	5.71(1.05)	6.28(1.33)	<0.001
FBG (mg/dl)	109.07(35.91)	107.88(34.61)	124.14(47.02)	<0.001
Smoke (%)				<0.001
Never	18034(55.2)	17132(56.5)	902(37.8)	
Past	7877(24.1)	6911(22.8)	966(40.5)	
Current	6772(20.7)	6256(20.6)	516(21.6)	
Drinking (%)				<0.001
No drinker	9645(29.5)	8922(29.4)	723(30.3)	
1-5drinks/month	16120(49.3)	14869(49.1)	1251(52.5)	
5-10drinks/month	2476(7.6)	2370(7.8)	106(4.4)	
10 + drinks/month	4442(13.6)	4138(13.7)	304(12.8)	
Follow-up time (month)	96.87(44.52)	98.11(44.28)	81.13(44.64)	<0.001
Log (PLR)	4.77(0.38)	4.77(0.38)	4.76(0.47)	0.222
Log (NLR)	0.67(0.47)	0.66(0.46)	0.83(0.53)	<0.001
Log (MLR)	−1.35(0.40)	−1.37(0.40)	−1.16(0.45)	<0.001
Log (SII)	6.15(0.55)	6.15(0.54)	6.20(0.62)	<0.001
Cardiovascular deaths (%)	949(2.9)	643(2.1)	306(12.8)	<0.001
All-cause deaths (%)	3805(11.6)	2935(9.7)	870(36.5)	<0.001

PIR, poverty income ratio; BMI, body mass index; TC, total cholesterol; TG, triglycerides; HDL-C, high-density lipoprotein cholesterol; LDL-C, low-density lipoprotein cholesterol; HbA1c, Glycosylated Hemoglobin type A1C; FBG, fasting blood glucose; PLR, platelet-to-lymphocyte ratio; NLR, neutrophil-to-lymphocyte ratio; MLR, monocyte-to-lymphocyte ratio; SII, the systemic immune inflammation index; CAD, coronary artery disease.

### The association between PLR, NLR, MLR, SII and CAD

The results of logistic regression analysis were shown in Table 2. Notably, NLR [OR: 1.29, 95%CI: 1.15–1.46, *P* < 0.001] and MLR [OR: 1.67, 95%CI: 1.40–1.99, *P* < 0.001] associated with increased risks of CAD when analyzed as continuous variables after adjusting for confounding factors, while PLR and SII did not demonstrate a significant association. Specifically, the highest MLR quartile (Q4) maintained a significant link to CAD in unadjusted model 1 [OR: 3.19, 95%CI: 2.65–3.83, *P* < 0.001], partially adjusted model 2 [OR: 1.61, 95%CI: 1.32–1.97, *P* < 0.001], and fully adjusted model 3 [OR: 1.68, 95%CI: 1.36–2.08, *P* < 0.001], in comparison to the lowest MLR quartile (Q1).

**Table 2:** Association of PLR, NLR, MLR, SII with the risk of CAD.

	Model1		Model2		Model3	
	*OR (95%CI)*	*P*	*OR (95%CI)*	*P*	*OR (95%CI)*	*P value*
Log (PLR)						
Q1: < 4.53	Ref		Ref		Ref	
Q2:4.53 ~ 4.77	0.81(0.71- 0.92)	0.002	0.85(0.75- 0.98)	0.021	0.92(0.80-1.06)	0.300
Q3:4.77 ~ 5.00	0.67(0.57- 0.80)	<0.001	0.70(0.59- 0.84)	<0.001	0.81(0.67-0.97)	0.025
Q4: > 5.00	0.87(0.76- 1.00)	0.058	0.78(0.67- 0.91)	0.002	0.91(0.78-1.08)	0.300
Continuous	0.86(0.73- 1.01)	0.066	0.82(0.72- 0.95)	0.007	0.94(0.81-1.09)	0.400
Log (NLR)						
Q1: < 0.38	Ref		Ref		Ref	
Q2: 0.38 ~ 0.67	1.00(0.84-1.19)	>0.900	0.92(0.76- 1.12)	0.400	0.91(0.75-1.10)	0.300
Q3: 0.67 ~ 0.96	1.14(0.97-1.34)	0.100	0.88(0.73- 1.06)	0.200	0.84(0.71-1.01)	0.061
Q4: > 0.96	2.06(1.75-2.43)	<0.001	1.18(0.98- 1.40)	0.081	1.09(0.91-1.29)	0.300
Continuous	2.15(1.89- 2.44)	<0.001	1.45(1.28- 1.63)	<0.001	1.29(1.15-1.46)	<0.001
Log (MLR)						
Q1: < −1.61	Ref		Ref		Ref	
Q2-1.61 ~ −1.39	1.13(0.92-1.39)	0.200	0.97(0.78-1.21)	0.800	1.04(0.84-1.30)	0.700
Q3: −1.39 ~ −1.10	1.54(1.27-1.87)	<0.001	1.11(0.90- 1.36)	0.300	1.17(0.94-1.45)	0.150
Q4: > −1.10	3.19(2.65-3.83)	<0.001	1.61(1.32-1.97)	<0.001	1.68(1.36-2.08)	<0.001
Continuous	3.49(2.96- 4.10)	<0.001	1.68(1.41- 2.00)	<0.001	1.67(1.40- 1.99)	<0.001
Log (SII)						
Q1: < 5.81	Ref		Ref		Ref	
Q2:5.81–6.15	0.80(0.68- 0.95)	0.012	0.79(0.66- 0.94)	0.009	0.77(0.64-0.93)	0.006
Q3:6.15–6.50	0.88(0.77- 1.00)	0.056	0.84(0.73- 0.96)	0.014	0.81(0.70-0.92)	0.002
Q4: > 6.50	1.14(0.98- 1.33)	0.091	1.00(0.86- 1.17)	>0.900	0.93(0.79-1.09)	0.400
Continuous	1.17(1.04- 1.32)	0.009	1.07(0.96- 1.20)	0.200	1.01(0.91- 1.13)	0.800

Model 1= unadjusted model (PLR/NLR/ MLR/SII).

Model 2= Model 1+ gender, age, race, PIR, education, BMI

Model 3 = Model 2 + HbA1c, FBG, TG, LDL-C, HDL-C, smoke, alcohol.

Odd ratios (ORs); Confidence Interval (CI); Q, quartiles; PIR, poverty income ratio; BMI, body mass index; HbA1c, Glycosylated Hemoglobin type A1C;FBG, fasting blood glucose; TG, triglycerides; LDL-C, low-density lipoprotein cholesterol; HDL-C, high-density lipoprotein cholesterol; PLR, platelet-to-lymphocyte ratio; NLR, neutrophil-to-lymphocyte ratio; MLR, monocyte-to-lymphocyte ratio; SII, the systemic immune inflammation index;CAD, coronary artery disease.

### The association between PLR, NLR, MLR, SII and all-cause mortality and cardiovascular mortality

**Fig 3:**
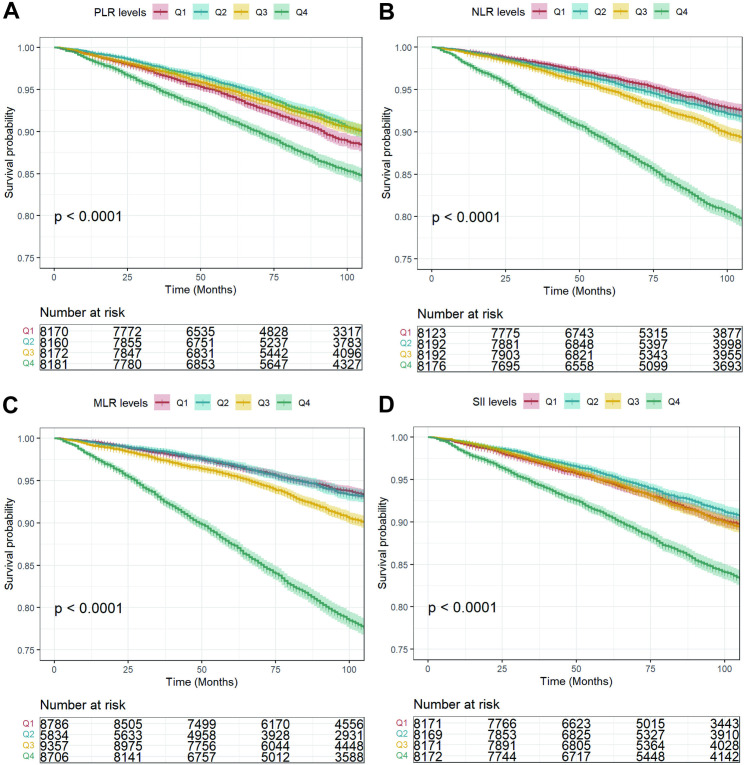
The Kaplan-Meier analysis of the prognostic effect of different levels of PLR, NLR, MLR and SII on all-cause mortality. The unit of time was month. PLR, platelet-to-lymphocyte ratio; NLR, neutrophil-to-lymphocyte ratio; MLR, monocyte-to-lymphocyte ratio; SII, the systemic immune inflammation index.

**Fig 4:**
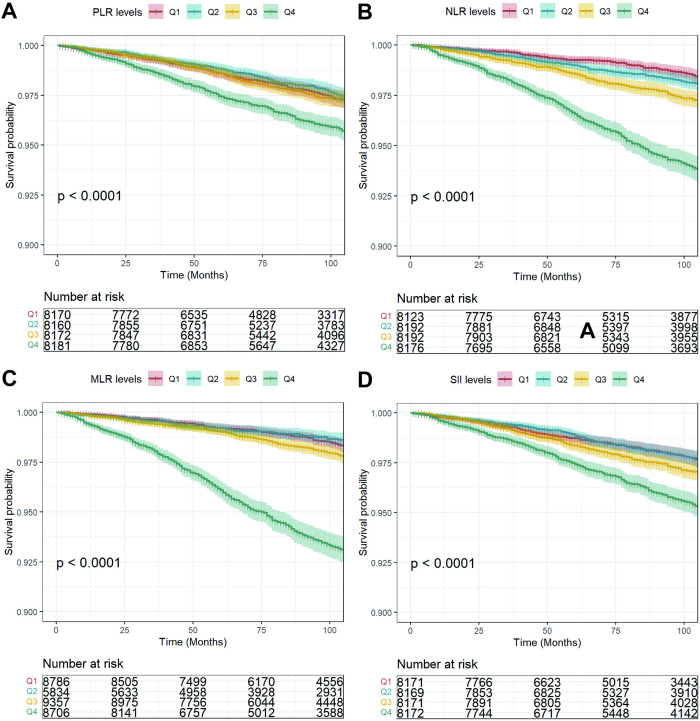
The Kaplan-Meier analysis of the prognostic effect of different levels of PLR, NLR, MLR and SII on cardiovascular mortality. The unit of time was month. PLR, platelet-to-lymphocyte ratio; NLR, neutrophil-to-lymphocyte ratio; MLR, monocyte-to-lymphocyte ratio; SII, the systemic immune inflammation index.

The Kaplan–Meier curve revealed an increasing trend in long-term all-cause mortality and cardiovascular mortality rates with higher levels of PLR, NLR, MLR, and SII (*P* < 0.001, Fig 3–4). Tables 3 and 4 further illustrated the association between PLR, NLR, MLR, and SII (both continuous and categorical) with increase risks of all-cause and cardiovascular mortality. After full adjustment, PLR (HR 1.24, 95% CI 1.08–1.42), NLR (HR 1.82, 95% CI 1.67–1.98), MLR (HR 2.32, 95% CI 2.09–2.58), and SII (HR 1.34, 95% CI 1.23–1.46) were positively linked to the risk of all-cause mortality. Similar results were shown when we categorized participants into quartiles by the PLR, NLR, MLR, and SII. We also observed a positively association between PLR (HR 1.77, 95% CI 1.41–2.23), NLR (HR 2.68, 95% CI 2.30–3.12), MLR (HR 3.55, 95% CI 2.96–4.26), SII (HR 1.79, 95% CI 1.53–2.08) and the risk of cardiovascular mortality.

**Table 3:** Association of PLR, NLR, MLR and SII with the risk of All-cause death.

	Model1		Model2		Model3	
	*OR (95%CI)*	*P*	*OR (95%CI)*	*P*	*OR (95%CI)*	*P*
Log (PLR)						
Q1: < 4.53	Ref		Ref		Ref	
Q2:4.53 ~ 4.77	0.75(0.66- 0.85)	<0.001	0.81(0.71- 0.92)	<0.001	0.84(0.74- 0.95)	0.005
Q3:4.77 ~ 5.00	0.75(0.66- 0.85)	<0.001	0.81(0.72- 0.91)	<0.001	0.88(0.77- 1.00)	0.043
Q4: > 5.00	1.13(1.01- 1.28)	0.038	1.04(0.93- 1.16)	0.500	1.13(1.00- 1.27)	0.046
Continuous	1.31(1.11- 1.54)	0.001	1.15(1.01- 1.31)	0.033	1.24(1.08- 1.42)	0.002
Log (NLR)						
Q1: < 0.38	Ref		Ref		Ref	
Q2: 0.38 ~ 0.67	1.07(0.93- 1.23)	0.400	1.06(0.92- 1.23)	0.400	1.05(0.91- 1.21)	0.500
Q3: 0.67 ~ 0.96	1.25(1.10- 1.43)	<0.001	1.11(0.97- 1.28)	0.120	1.09(0.96- 1.24)	0.200
Q4: > 0.96	2.68(2.39- 3.01)	<0.001	1.98(1.78- 2.20)	<0.001	1.88(1.69- 2.11)	<0.001
Continuous	2.64(2.40- 2.90)	<0.001	1.91(1.76- 2.07)	<0.001	1.82(1.67- 1.98)	<0.001
Log (MLR)						
Q1: < −1.61	Ref		Ref		Ref	
Q2-1.61 ~ −1.39	1.16(1.02- 1.33)	0.022	0.97(0.83- 1.14)	0.700	1.02(0.87- 1.19)	0.800
Q3: −1.39 ~ −1.10	1.58(1.39- 1.81)	<0.001	1.17(1.03- 1.33)	0.015	1.23(1.08- 1.40)	0.002
Q4: > −1.10	3.33(2.95- 3.75)	<0.001	1.82(1.60- 2.06)	<0.001	1.91(1.68- 2.18)	<0.001
Continuous	4.26(3.81- 4.77)	<0.001	2.27(2.04- 2.52)	<0.001	2.32(2.09- 2.58)	<0.001
Log (SII)						
Q1: < 5.81	Ref		Ref		Ref	
Q2:5.81–6.15	0.85(0.75- 0.98)	0.024	0.87(0.76- 1.00)	0.047	0.87(0.75- 1.00)	0.055
Q3:6.15–6.50	0.92(0.80- 1.05)	0.200	0.93(0.81- 1.06)	0.300	0.92(0.80- 1.05)	0.200
Q4: > 6.50	1.55(1.38- 1.74)	<0.001	1.46(1.31- 1.64)	<0.001	1.42(1.26- 1.60)	<0.001
Continuous	1.47(1.33- 1.63)	<0.001	1.38(1.27- 1.51)	<0.001	1.34(1.23- 1.46)	<0.001

Model 1= unadjusted model (PLR/NLR/ MLR/SII).

Model 2= Model 1+ gender, age, race, PIR, education, BMI

Model 3 = Model 2 + HbA1c, FBG, TG, LDL-C, HDL-C, smoke, alcohol.

hazard ratios (HRs); Confidence Interval (CI); Q, quartiles; PIR, poverty income ratio; BMI, body mass index; HbA1c, Glycosylated Hemoglobin type A1C;FBG, fasting blood glucose; TG, triglycerides; LDL-C, low-density lipoprotein cholesterol; HDL-C, high-density lipoprotein cholesterol; PLR, platelet-to-lymphocyte ratio; NLR, neutrophil-to-lymphocyte ratio; MLR, monocyte-to-lymphocyte ratio; SII, the systemic immune inflammation index.

**Table 4:** Association of PLR, NLR, MLR and SII with the risk of Cardiovascular deaths.

	Model1		Model2		Model3	
	*HR (95%CI)*	*P*	*HR (95%CI)*	*P*	*HR (95%CI)*	*P*
Log (PLR)						
Q1: < 4.53	Ref		Ref		Ref	
Q2:4.53 ~ 4.77	0.77(0.58- 1.01)	0.062	0.84(0.64- 1.11)	0.200	0.88(0.67- 1.16)	0.400
Q3:4.77 ~ 5.00	0.90(0.70- 1.15)	0.400	1.01(0.77- 1.32)	>0.900	1.10(0.83- 1.44)	0.500
Q4: > 5.00	1.43(1.15- 1.76)	<0.001	1.36(1.09- 1.69)	0.006	1.48(1.18- 1.86)	<0.001
Continuous	1.91(1.49- 2.44)	<0.001	1.64(1.30- 2.06)	<0.001	1.77(1.41- 2.23)	<0.001
Log (NLR)						
Q1: < 0.38	Ref		Ref		Ref	
Q2: 0.38 ~ 0.67	1.09(0.79- 1.50)	0.600	1.11(0.81- 1.52)	0.500	1.10(0.80- 1.52)	0.600
Q3: 0.67 ~ 0.96	1.75(1.28- 2.40)	<0.001	1.57(1.15- 2.15)	0.004	1.54(1.14- 2.08)	0.005
Q4: > 0.96	4.16(3.18- 5.45)	<0.001	3.05(2.34- 3.99)	<0.001	2.94(2.25- 3.83)	<0.001
Continuous	3.96(3.36- 4.66)	<0.001	2.77(2.38- 3.22)	<0.001	2.68(2.30- 3.12)	<0.001
Log (MLR)						
Q1: < −1.61	Ref		Ref		Ref	
Q2-1.61 ~ −1.39	0.89(0.65- 1.23)	0.500	0.78(0.57- 1.07)	0.130	0.81(0.59- 1.11)	0.200
Q3: −1.39 ~ −1.10	1.51(1.10- 2.07)	0.010	1.13(0.83- 1.55)	0.400	1.19(0.87- 1.62)	0.300
Q4: > −1.10	4.35(3.32- 5.69)	<0.001	2.28(1.77- 2.94)	<0.001	2.39(1.87- 3.06)	<0.001
Continuous	6.56(5.39- 7.98)	<0.001	3.49(2.89- 4.21)	<0.001	3.55(2.96- 4.26)	<0.001
Log (SII)						
Q1: < 5.81	Ref		Ref		Ref	
Q2:5.81–6.15	0.92(0.73- 1.16)	0.500	0.94(0.76- 1.18)	0.600	0.95(0.76- 1.19)	0.600
Q3:6.15–6.50	1.16(0.91- 1.48)	0.200	1.20(0.93- 1.53)	0.200	1.20(0.94- 1.53)	0.200
Q4: > 6.50	1.99(1.64- 2.42)	<0.001	1.91(1.55- 2.34)	<0.001	1.90(1.54- 2.33)	<0.001
Continuous	1.97(1.68- 2.30)	<0.001	1.81(1.56- 2.11)	<0.001	1.79(1.53- 2.08)	<0.001

Model 1= unadjusted model (PLR/NLR/ MLR/SII).

Model 2= Model 1+ gender, age, race, PIR, education, BMI

Model 3 = Model 2 + HbA1c, FBG, TG, LDL-C, HDL-C, smoke, alcohol.

hazard ratios (HRs); Confidence Interval (CI); Q, quartiles; PIR, poverty index; BMI, body mass index; HbA1c, Glycosylated Hemoglobin type A1C;TG, triglycerides; FBG, fasting blood glucose; LDL-C, low-density lipoprotein cholesterol; HDL-C, high-density lipoprotein cholesterol; PLR, platelet-to-lymphocyte ratio; NLR, neutrophil-to-lymphocyte ratio; MLR, monocyte-to-lymphocyte ratio; SII, the systemic immune inflammation index.

### Restricted cubic splines analysis

**Fig 5:**
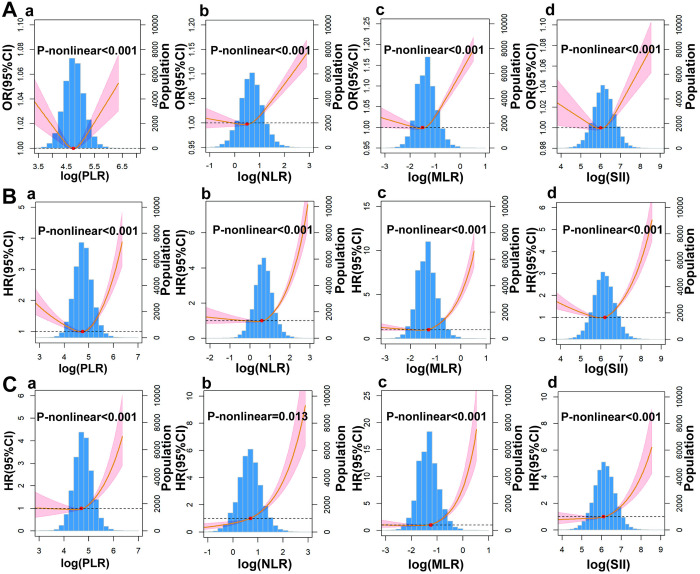
(A) Association between PLR, NLR, MLR, SII and CAD; (B) Association between PLR, NLR, MLR, SII and all-cause mortality; (C) Association between PLR, NLR, MLR, SII and cardiovascular mortality; (a)PLR; (b)NLR; (c)MLR; (d) SII. PLR, platelet-to-lymphocyte ratio; NLR, neutrophil-to-lymphocyte ratio; MLR, monocyte-to-lymphocyte ratio; SII, the systemic immune inflammation index; CAD, coronary artery diseases.

The RCS analysis revealed a U-shaped association between PLR and SII with CAD, whereas NLR and MLR exhibited a J-shaped relationship (Fig 5). Segmented regression and threshold analysis (Table 5) identified an inflection point for log (PLR) at 4.93(PLR = 138.38). When log (PLR) exceeded this value, the risk of CAD increased markedly (OR = 1.61, 95% CI:1.26–2.04, *P* < 0.001). Similarly, when log (SII) exceeded 6.11(SII = 450.34), the risk of CAD increased by 34% (OR = 1.34, 95% CI: 1.17–1.52, *P* < 0.001). Furthermore, PLR, NLR, MLR, and SII all exhibited a J-shaped relationship with both all-cause and cardiovascular mortality (*P* for nonlinearity > 0.05).

**Table 5:** The threshold effect of PLR, NLR, MLR and SII on CAD was analyzed using a two-stage phased regression model.

Models	Adjusted OR (95%CI)	*P* value
Log (PLR)		
logistic regression model I	0.94(0.81,1.09)	0.400
Model II		
Inflection point		
< 4.93(PLR = 138.38)	0.80 (0.69,0.93)	0.003
> 4.93(PLR = 138.38)	1.61(1.26,2.04)	<0.001
Log likelihood ratio		<0.001
Log (NLR)		
logistic regression model I	1.28(1.13,1.44)	<0.001
Model II		
Inflection point		
<0.73(NLR = 2.08)	1.06 (0.89,1.27)	0.506
>0.73(NLR = 2.08)	1.67 (1.44,1.94)	<0.001
Log likelihood ratio		0.001
Log (MLR)		
logistic regression model I	1.67(1.40,1.99)	<0.001
Model II		
Inflection point		
<−1.74(MLR = 0.18)	0.64(0.46,0.92)	0.008
> −1.74(MLR = 0.18)	1.96(1.73,2.22)	<0.001
Log likelihood ratio		<0.001
Log (SII)		
logistic regression model I	1.01(0.91,1.13)	0.800
Model II		
Inflection point		
< 6.11(SII = 450.34)	0.86 (0.75,0.99)	0.039
> 6.11(SII = 450.34)	1.34(1.17,1.52)	<0.001
Log likelihood ratio		<0.001

Odd ratios (ORs); PLR, platelet-to-lymphocyte ratio; NLR, neutrophil-to-lymphocyte ratio; MLR, monocyte-to-lymphocyte ratio; SII, the systemic immune inflammation index;CAD, coronary artery disease.

### Diagnostic performance of four inflammatory markers for CAD

This study compared the AUC values of four inflammatory markers to distinguish CAD (Table 6), and found that MLR had the highest AUC value (AUC:0.642, 95% CI: 0.629–0.654), followed by NLR (AUC:0.600, 95% CI: 0.587–0.612).

**Table 6:** AUCs of four inflammation indexes for discriminating CAD.

Variables	AUC (95%CI)	Cutoff points	Youden’s index	Sensitivity (%)	Specificity (%)
PLR	0.506(0.493-0.519)	88.44	0.042	24.41	79.79
NLR	0.600(0.587-0.612)	2.28	0.157	50.25	65.46
MLR	0.642(0.629-0.654)	0.30	0.222	54.82	67.33
SII	0.530(0.518,0.543)	675.29	0.058	29.40	76.36

PLR, platelet-to-lymphocyte ratio;NLR, neutrophil-to-lymphocyte ratio; MLR, monocyte-to-lymphocyte ratio; SII, the systemic immune inflammation index; CAD, coronary artery disease.

### Sensitivity analyses

In the sensitivity analysis, the results were consistent after excluding participants with missing data (Supplementary Table 1). The analysis revealed a positive association between NLR, MLR, and the risk of CAD. Moreover, the relationships between PLR, NLR, MLR, SII, and CAD were reaffirmed in 10 imputed datasets (Supplementary Table 2), with findings aligning with those of prior studies.

## Discussion

The study indicated a positive link between NLR and MLR with CAD. Notably, PLR and SII exhibited a U-shaped association with the risk of CAD, with a marked escalation in risk occurring once these markers surpassed certain thresholds. Higher levels of PLR, NLR, MLR, and SII were predictive of an increased risk of all-cause and cardiovascular mortality, regardless of whether they were analyzed continuously or as categories. Additionally, compared with PLR and SII, NLR and MLR had the better discriminating ability for CAD. In general, these findings emphasize the need for further investigation into the potential utility of PLR, NLR, MLR, and SII in preventing or reducing the effects of CAD, as well as indicating that PLR, NLR, MLR, and SII may be useful prognostic markers for all-cause and cardiovascular mortality.

Many studies have demonstrated a growing interest in utilizing inflammatory biomarkers to investigate their association with CAD. While common biomarkers such as CRP, IL-1β, and IL-6 indicate acute inflammation, they may not fully capture chronic inflammation levels [18]. Therefore, a series of composite biomarkers from peripheral blood, including PLR, NLR, MLR, and SII, can provide a more comprehensive judgment of the body’s inflammatory levels by analyzing platelets, neutrophils, and lymphocytes. Recent research has explored the link between CAD and composite inflammatory biomarkers in individuals with a chronic inflammation-related disease. For instance, Zhai et al.’s study revealed that MLR was predictive of a higher risk of cardiovascular mortality in peritoneal dialysis individuals [19]. Similarly, Yin et al.’s study demonstrated that NLR independently predicted CAD and all-cause mortality in patients with rheumatoid arthritis [20]. Additionally, a study by Liu and colleagues emphasized that MLR was a significant independent predictor of mortality and cardiovascular mortality in individuals with chronic kidney diseases [21]. However, there is a lack of data on the significance of these inflammatory parameters (PLR, NLR, MLR, and SII) in the general population. Chronic inflammation may manifest in healthy individuals, while chronic inflammation may not cause chronic diseases, individuals experiencing chronic inflammation are at a higher risk of developing chronic diseases like CAD. The findings in this study indicated that high levels of PLR, NLR, MLR and SII were linked to an increased prevalence of CAD, and high levels of these biomarkers were also predictive of a higher risk of all-cause and cardiovascular mortality in the general population, providing evidence to better identify chronic inflammation levels in the general population and implement timely preventive measures to mitigate the impact of CAD.

In this study, PLR, NLR, MLR and SII were found to be nonlinearly associated with CAD. Specifically, CAD risk rised sharply when inflammation levels exceeded a certain threshold. The relationship between PLR, NLR, MLR and SII with CAD may be explained by the interaction of neutrophils, monocytes, lymphocytes, and platelets in the immune response. The progression of CAD is closely linked to the activation of inflammatory and immune responses [22]. Prolonged exposure to certain risk factors, such as hyperlipidemia, hyperglycemia, and smoking, can result in endothelial dysfunction [23], which triggers an inflammatory response. Neutrophils, a type of white blood cell, play a key role in this process by containing pathogens and activating adaptive immunity [24,25]. However, in an inflammatory environment, neutrophils can also release harmful reactive oxygen species that damage the blood vessel walls and form neutrophil extracellular traps (NETs) that may contribute to atheromatous plaque formation or plaque rupture. [26]. Additionally, neutrophils can promote the recruitment of other inflammatory cells and activated platelets, further enhancing the inflammatory state [27,28]. Monocytes, another type of immune cell, are pro-inflammatory cells that produce cytokines, chemokines, and reactive oxygen species. In CAD, monocytes are crucial in absorbing oxidized lipids and transforming into foam cells, which form the core of atherosclerotic plaques in the arteries [29,30]. Subsequently, monocytes release pro-inflammatory cytokines and enzymes that can exacerbate atherosclerosis. Apart from neutrophils and monocytes, lymphocytes are immune cells that may have protective or pro-atherogenic roles in CAD. Th1 cells contribute to atherosclerosis by promoting endothelial dysfunction and lipid accumulation in macrophages, while regulatory T cells (Tregs) have a protective effect by promoting immune tolerance and reducing inflammation within the arterial wall [31,32]. Therefore, the impact of lymphocytes on CAD outcomes depends on the specific subsets. Overall, studies have shown that a decreased lymphocyte count is associated with worse cardiovascular outcomes in individuals with CAD and heart failure [33].The association between inflammatory indicators and CAD is thought to arise from a complex interplay among immune cells. NLR, MLR and SII are considered indicators of immune and inflammatory responses, whereas PLR represents the relationship between thrombotic and inflammatory conditions. SII integrates multiple blood cell parameters, providing a more comprehensive reflection of systemic and whole-body inflammatory status.

PLR, NLR, MLR, and SII were associated with an increased prevalence of CAD and mortality risk in the general population, not only reflecting statistical associations but also emphasizing the clinical significance of our findings. These readily available inflammatory indices hold significant clinical implications for the prevention and management of CAD. As routine blood test-derived parameters, PLR, NLR, MLR and SII offer cost-effective tools for risk stratification in primary care settings. Their elevation may prompt earlier referral for specialized cardiac evaluation, particularly in asymptomatic high-risk individuals. The integration of these indices with traditional risk factors could enhance the predictive accuracy of current risk assessment models. Specifically, those showing high levels of PLR, NLR, MLR and SII could be prioritized for enhanced cardiovascular risk management, including thorough monitoring, lifestyle modifications, and intensified pharmacological interventions to mitigate their increased risk of mortality.

This study has several notable strengths and limitations. Utilizing a nationwide, large-scale cohort enabled a comprehensive analysis of the associations between inflammatory markers and a range of outcomes. Converting independent variables into quartiles as categorical variables helped mitigate the impact of outliers and skewed data distributions, thereby enhancing the robustness of the model results. Additionally, RCS were employed to explore the non-linear relationships of PLR, NLR, and MLR with CAD, all-cause mortality, and cardiovascular mortality, while segmented regression and threshold analysis were conducted to refine discriminatory accuracy. Notably, our discoveries augment the growing body of literature on the prognostic importance of inflammatory biomarkers in CAD and survival outcomes within the general population. Grasping these mechanisms is vital for devising strategies focused on preventing and managing CAD by targeting inflammation and immune responses.

Nevertheless, there are still some limitations. Firstly, this study is a retrospective longitudinal cohort study and cannot fully control for the temporal relationship between exposure and outcome. Although an association between exposure and outcome was observed, causal inference remains limited due to potential confounding factors such as genetic factors and diet, reverse causality, or omitted variables. This underscores the necessity for future large-scale, multicenter, prospective cohort studies to validate these findings. Secondly, caution must be exercised when interpreting the results due to the potential use of anti-inflammatory medications for other conditions among certain patients. Additionally, the lack of data on cardiovascular disease-specific medication use may have affected the findings. Thirdly, self-reported CAD diagnoses may lead to the omission of some undiagnosed CAD patients. Furthermore, the findings, which are based on American adults, may not be generalizable to other races, regions, and countries. Further multi-center research is needed in the future. Lastly, measuring PLR, NLR, MLR, and SII at just one time point may not adequately reflect potential variations over time or in response to treatment. Multiple measurements over time could yield a more comprehensive understanding of the inflammatory process.

## Conclusion

This research demonstrated positive associations between platelet-to-lymphocyte ratio, neutrophil-to-lymphocyte ratio, monocyte-to-lymphocyte ratio, and systemic immune inflammation index with coronary artery disease, all-cause and cardiovascular mortality. The strong association between these inflammatory markers and CAD prevalence supports their potential utility as adjunctive biomarkers in clinical practice. Future studies should validate optimal cutoff values and evaluate whether intervention strategies guided by these indices can improve patient outcomes.

## Supporting information

Supplementary Table 1 The association between PLR, NLR, MLR, SII with the risk of CAD after excluding participants with missing data. (DOCX)

Supplementary Table 2 The association between PLR, NLR, MLR, SII with the risk of CAD for multiple imputations (10 times). (DOCX)

S1 Data COMPLETED.DATA20241210 (CSV)

## References

[1] Roth GA, Mensah GA, Johnson CO, Addolorato G, Ammirati E, Baddour LM, et al. Global burden of cardiovascular diseases and risk factors, 1990-2019: update from the GBD 2019 study. J Am Coll Cardiol. 2020;76(25):2982–3021. doi: 10.1016/j.jacc.2020.11.010 33309175 PMC7755038

[2] Writing Group Members, Mozaffarian D, Benjamin EJ, Go AS, Arnett DK, Blaha MJ, et al. Heart disease and stroke statistics-2016 update: a report from the American Heart Association. Circulation. 2016;133(4):e38–360. doi: 10.1161/CIR.0000000000000350 26673558

[3] Henein MY, Vancheri S, Longo G, Vancheri F. The role of inflammation in cardiovascular disease. Int J Mol Sci. 2022;23(21):12906. doi: 10.3390/ijms232112906 36361701 PMC9658900

[4] Ferrucci L, Fabbri E. Inflammageing: chronic inflammation in ageing, cardiovascular disease, and frailty. Nat Rev Cardiol. 2018;15(9):505–22. doi: 10.1038/s41569-018-0064-2 30065258 PMC6146930

[5] Alfaddagh A, Martin SS, Leucker TM, Michos ED, Blaha MJ, Lowenstein CJ, et al. Inflammation and cardiovascular disease: From mechanisms to therapeutics. Am J Prev Cardiol. 2020;4:100130. doi: 10.1016/j.ajpc.2020.100130 34327481 PMC8315628

[6] Adamo L, Rocha-Resende C, Prabhu SD, Mann DL. Reappraising the role of inflammation in heart failure. Nat Rev Cardiol. 2020;17(5):269–85. doi: 10.1038/s41569-019-0315-x 31969688

[7] Leuti A, Fazio D, Fava M, Piccoli A, Oddi S, Maccarrone M. Bioactive lipids, inflammation and chronic diseases. Adv Drug Deliv Rev. 2020;159:133–69. doi: 10.1016/j.addr.2020.06.028 32628989

[8] Haybar H, Shokuhian M, Bagheri M, Davari N, Saki N. Involvement of circulating inflammatory factors in prognosis and risk of cardiovascular disease. J Mol Cell Cardiol. 2019;132:110–9. doi: 10.1016/j.yjmcc.2019.05.010 31102585

[9] Jaén RI, Val-Blasco A, Prieto P, Gil-Fernández M, Smani T, López-Sendón JL, et al. Innate immune receptors, key actors in cardiovascular diseases. JACC Basic Transl Sci. 2020;5(7):735–49. doi: 10.1016/j.jacbts.2020.03.015 32760860 PMC7393405

[10] Dong M, Shi Y, Yang J, Zhou Q, Lian Y, Wang D, et al. Prognostic and clinicopathological significance of systemic immune-inflammation index in colorectal cancer: a meta-analysis. Ther Adv Med Oncol. 2020;12:1758835920937425. doi: 10.1177/1758835920937425 32699557 PMC7357045

[11] Chen L, Kong X, Wang Z, Wang X, Fang Y, Wang J. Pre-treatment systemic immune-inflammation index is a useful prognostic indicator in patients with breast cancer undergoing neoadjuvant chemotherapy. J Cell Mol Med. 2020;24(5):2993–3021. doi: 10.1111/jcmm.14934 31989747 PMC7077539

[12] Libby P. The changing landscape of atherosclerosis. Nature. 2021;592(7855):524–33. doi: 10.1038/s41586-021-03392-8 33883728

[13] Christensen K, Gleason CE, Mares JA. Dietary carotenoids and cognitive function among US adults, NHANES 2011-2014. Nutr Neurosci. 2020;23(7):554–62. doi: 10.1080/1028415X.2018.1533199 30326796 PMC6467741

[14] Hrubaru I, Motoc A, Moise ML, Miutescu B, Citu IM, Pingilati RA, et al. The predictive role of maternal biological markers and inflammatory scores NLR, PLR, MLR, SII, and SIRI for the risk of preterm delivery. J Clin Med. 2022;11(23):6982. doi: 10.3390/jcm11236982 36498555 PMC9738289

[15] Paulose-Ram R, Graber JE, Woodwell D, Ahluwalia N. The National Health and Nutrition Examination Survey (NHANES), 2021-2022: adapting data collection in a COVID-19 environment. Am J Public Health. 2021;111(12):2149–56. doi: 10.2105/AJPH.2021.306517 34878854 PMC8667826

[16] Liu C, Liang D, Xiao K, Xie L. Association between the triglyceride-glucose index and all-cause and CVD mortality in the young population with diabetes. Cardiovasc Diabetol. 2024;23(1):171. doi: 10.1186/s12933-024-02269-0 38755682 PMC11097545

[17] Kim JH. Multicollinearity and misleading statistical results. Korean J Anesthesiol. 2019;72(6):558–69. doi: 10.4097/kja.19087 31304696 PMC6900425

[18] Rasmussen LJH, Petersen JEV, Eugen-Olsen J. Soluble urokinase plasminogen activator receptor (suPAR) as a biomarker of systemic chronic inflammation. Front Immunol. 2021;12:780641. doi: 10.3389/fimmu.2021.780641 34925360 PMC8674945

[19] Yang Y, Xu Y, Lu P, Zhou H, Yang M, Xiang L. The prognostic value of monocyte-to-lymphocyte ratio in peritoneal dialysis patients. Eur J Med Res. 2023;28(1):152. doi: 10.1186/s40001-023-01073-y 37038225 PMC10084613

[20] Zhou E, Wu J, Zhou X, Yin Y. The neutrophil-lymphocyte ratio predicts all-cause and cardiovascular mortality among U.S. adults with rheumatoid arthritis: results from NHANES 1999-2020. Front Immunol. 2023;14:1309835. doi: 10.3389/fimmu.2023.1309835 38045692 PMC10690944

[21] Liu W, Weng S, Cao C, Yi Y, Wu Y, Peng D. Association between monocyte-lymphocyte ratio and all-cause and cardiovascular mortality in patients with chronic kidney diseases: A data analysis from national health and nutrition examination survey (NHANES) 2003-2010. Ren Fail. 2024;46(1):2352126. doi: 10.1080/0886022X.2024.2352126 38832474 PMC11151800

[22] Osborn O, Olefsky JM. The cellular and signaling networks linking the immune system and metabolism in disease. Nat Med. 2012;18(3):363–74. doi: 10.1038/nm.2627 22395709

[23] Domingo E, Marques P, Francisco V, Piqueras L, Sanz M-J. Targeting systemic inflammation in metabolic disorders. A therapeutic candidate for the prevention of cardiovascular diseases?. Pharmacol Res. 2024;200:107058. doi: 10.1016/j.phrs.2024.107058 38218355

[24] Rosales C. Neutrophils at the crossroads of innate and adaptive immunity. J Leukoc Biol. 2020;108:377–96. doi: 10.1002/jlb.4mir0220-574rr . 32202340

[25] Gaul DS, Stein S, Matter CM. Neutrophils in cardiovascular disease. Eur Heart J. 2017;38:1702–4. doi: 10.1093/eurheartj/ehx244 30052884

[26] Döring Y, Libby P, Soehnlein O. Neutrophil extracellular traps participate in cardiovascular diseases: recent experimental and clinical insights. Circ Res. 2020;126(9):1228–41. doi: 10.1161/CIRCRESAHA.120.315931 32324499 PMC7185047

[27] Ramirez GA, Manfredi AA, Maugeri N. Misunderstandings between platelets and neutrophils build in chronic inflammation. Front Immunol. 2019;10:2491. doi: 10.3389/fimmu.2019.02491 31695699 PMC6817594

[28] Totani L, Evangelista V. Platelet-leukocyte interactions in cardiovascular disease and beyond. Arterioscler Thromb Vasc Biol. 2010;30(12):2357–61. doi: 10.1161/ATVBAHA.110.207480 21071701 PMC3076621

[29] Yang J, Zhang L, Yu C, Yang X-F, Wang H. Monocyte and macrophage differentiation: circulation inflammatory monocyte as biomarker for inflammatory diseases. Biomark Res. 2014;2(1):1. doi: 10.1186/2050-7771-2-1 24398220 PMC3892095

[30] Kim K-W, Ivanov S, Williams JW. Monocyte recruitment, specification, and function in atherosclerosis. Cells. 2020;10(1):15. doi: 10.3390/cells10010015 33374145 PMC7823291

[31] Sharma M, Schlegel MP, Afonso MS, Brown EJ, Rahman K, Weinstock A, et al. Regulatory T cells license macrophage pro-resolving functions during atherosclerosis regression. Circ Res. 2020;127(3):335–53. doi: 10.1161/CIRCRESAHA.119.316461 32336197 PMC7367765

[32] Wang X, Zhou H, Liu Q, Cheng P, Zhao T, Yang T, et al. Targeting regulatory T cells for cardiovascular diseases. Front Immunol. 2023;14:1126761. doi: 10.3389/fimmu.2023.1126761 36911741 PMC9995594

[33] Núñez J, Miñana G, Bodí V, Núñez E, Sanchis J, Husser O, et al. Low lymphocyte count and cardiovascular diseases. Curr Med Chem. 2011;18(21):3226–33. doi: 10.2174/092986711796391633 21671854

