# Supplementary material for: A retrospective analysis from NHANES 2003–2018 on the associations between inflammatory markers and coronary artery disease, all-cause mortality and cardiovascular mortality
Source: PLoS One. 2025 Jul 9;20(7):e0326953. doi: 10.1371/journal.pone.0326953 (PMC12240292; doi:10.1371/journal.pone.0326953)
Supplement: Supplementary Table 1 — (DOCX) [file pone.0326953.s001.docx]

Supplementary Table 1. The association between PLR, NLR, MLR, SII with the risk of CAD after excluding participants with missing data.

|  | Model1 | | Model2 | | Model3 | |
| --- | --- | --- | --- | --- | --- | --- |
|  | *OR (95%CI)* | *P* | *OR (95%CI)* | *P* | *OR (95%CI)* | *P* |
| Log (PLR) | 0.80(0.60- 1.08) | 0.150 | 0.78(0.61- 1.02) | 0.067 | 0.87(0.66- 1.14) | 0.300 |
| Log (NLR) | 2.48(1.98- 3.11) | <0.001 | 1.54(1.24- 1.91) | <0.001 | 1.30(1.06- 1.60) | 0.013 |
| Log (MLR) | 3.43(2.66- 4.43) | <0.001 | 1.53(1.17- 2.00) | 0.002 | 1.51(1.14- 1.99) | 0.004 |
| Log (SII) | 1.39(1.13- 1.71) | 0.002 | 1.21(1.02- 1.45) | 0.032 | 1.10(0.92- 1.30) | 0.300 |

Model 1= unadjusted model (PLR/NLR/ MLR/SII).

Model 2= Model 1+ gender, age, race, PIR, education, BMI

Model 3= Model 2+ HbA1c, FBG, TG, LDL-C, HDL-C, smoke, alcohol.

Odd ratios (ORs); Confidence Interval (CI); Q, quartiles; PIR, poverty index; BMI, body mass index; FBG, fasting blood glucose; TG, triglycerides; LDL-C, low-density lipoprotein cholesterol; HDL-C, high-density lipoprotein cholesterol; PLR, platelet-to-lymphocyte ratio; NLR, neutrophil-to-lymphocyte ratio; MLR, monocyte-to-lymphocyte ratio; SII, the systemic immune inflammation index.
