# Supplementary material for: A retrospective analysis from NHANES 2003–2018 on the associations between inflammatory markers and coronary artery disease, all-cause mortality and cardiovascular mortality
Source: PLoS One. 2025 Jul 9;20(7):e0326953. doi: 10.1371/journal.pone.0326953 (PMC12240292; doi:10.1371/journal.pone.0326953)
Supplement: Supplementary Table 2 — (DOCX) [file pone.0326953.s002.docx]

Supplementary Table 2 The association between PLR, NLR, MLR, SII with the risk of CAD for multiple imputations (10 times)

| Multiple imputation | PLR | | NLR | | MLR | | SII | |
| --- | --- | --- | --- | --- | --- | --- | --- | --- |
|  | OR (95%CI) | *P* | OR (95%CI) | *P* | OR (95%CI) | *P* | OR (95%CI) | *P* |
| 1 | 0.94(0.81- 1.09) | 0.400 | 1.29(1.15- 1.46) | <0.001 | 1.67(1.40- 1.99) | <0.001 | 1.01(0.91- 1.13) | 0.800 |
| 2 | 0.93(0.80- 1.08) | 0.300 | 1.30(1.15- 1.46) | <0.001 | 1.67(1.40- 2.00) | <0.001 | 1.02(0.92- 1.14) | 0.700 |
| 3 | 0.93(0.80- 1.08) | 0.300 | 1.29(1.14- 1.45) | <0.001 | 1.66(1.39- 1.98) | <0.001 | 1.01(0.90- 1.12) | 0.900 |
| 4 | 0.93(0.80- 1.08) | 0.300 | 1.29(1.15- 1.46) | <0.001 | 1.67(1.40- 1.99) | <0.001 | 1.01(0.91- 1.13) | 0.800 |
| 5 | 0.93(0.80- 1.08) | 0.300 | 1.30(1.15- 1.46) | <0.001 | 1.66(1.39- 1.98) | <0.001 | 1.01(0.91- 1.12) | 0.900 |
| 6 | 0.94(0.81- 1.09) | 0.400 | 1.29(1.15- 1.46) | <0.001 | 1.67(1.40- 1.99) | <0.001 | 1.01(0.91- 1.13) | 0.800 |
| 7 | 0.93(0.80- 1.08) | 0.300 | 1.30(1.15- 1.46) | <0.001 | 1.67(1.40- 2.00) | <0.001 | 1.02(0.92- 1.14) | 0.700 |
| 8 | 0.93(0.80- 1.08) | 0.300 | 1.29(1.14- 1.45) | <0.001 | 1.66(1.39- 1.98) | <0.001 | 1.01(0.90- 1.12) | 0.900 |
| 9 | 0.93(0.80- 1.08) | 0.300 | 1.28(1.13- 1.44) | <0.001 | 1.67(1.40- 1.99) | <0.001 | 1.01(0.91- 1.13) | 0.800 |
| 10 | 0.93(0.80- 1.08) | 0.400 | 1.30(1.15- 1.46) | <0.001 | 1.66(1.39- 1.98) | <0.001 | 1.01(0.91- 1.12) | 0.900 |

Models adjusted age, gender, race, education level, PIR, BMI, HbA1c, FBG, TG, LDL-C, HDL-C, smoke, alcohol.

Odd ratios (ORs); Confidence Interval (CI); Q, quartiles; PIR, poverty index; BMI, body mass index; FBG, fasting blood glucose; TG, triglycerides; LDL-C, low-density lipoprotein cholesterol; HDL-C, high-density lipoprotein cholesterol; PLR, platelet-to-lymphocyte ratio; NLR, neutrophil-to-lymphocyte ratio; MLR, monocyte-to-lymphocyte ratio; SII, the systemic immune inflammation index.
